# Towards eco-friendly apple farming: Real-time codling moth monitoring using improved YOLOv10 and IoT integration

**DOI:** 10.1371/journal.pone.0346415

**Published:** 2026-04-15

**Authors:** Mohamed Zarboubi, Abdelaaziz Bellout, Samira Chabaa, Azzedine Dliou

**Affiliations:** 1 LISAD Laboratory, National School of Applied Sciences, Ibn Zohr University, Agadir, Morocco; 2 LISTI Laboratory, National School of Applied Sciences, Ibn Zohr University, Agadir, Morocco; 3 I2SP Team, Faculty of Sciences Semlalia, Cadi Ayyad University, Marrakech, Morocco; 4 IMIS Laboratory, Faculty of Applied Sciences, Ibn Zohr University, Agadir, Morocco; Sher-e Kashmir University of Agricultural Sciences and Technology of Jammu, INDIA

## Abstract

Pest-related crop losses pose a critical threat to food security and sustainable agriculture, especially in apple orchards where the codling moth (Cydia pomonella) is a major concern. This study introduces an advanced pest monitoring system that integrates an improved YOLOv10-m deep learning model with Internet of Things (IoT) technology, designed specifically for real-time detection of codling moths. The system operates on a low-power Raspberry Pi platform, making it accessible and cost-effective for widespread field deployment. By enabling precise, geolocated, and real-time monitoring of pest populations, the system facilitates the rational and timely application of pesticides—only when and where they are truly needed. This not only enhances the effectiveness of pest control but also significantly reduces excessive chemical usage, thereby minimizing harmful residues in the environment and promoting better human health outcomes. Comparative evaluation against YOLO versions 5–12 confirms the superior balance of accuracy, confidence stability, and computational efficiency of the proposed model. Aligned with the principles of Integrated Pest Management (IPM), this approach promotes eco-friendly and health-conscious farming practices. Ultimately, the study demonstrates the potential of combining AI and IoT technologies to revolutionize pest management, contributing to a more sustainable and responsible agricultural ecosystem.

## 1. Introduction

Apple ranks as one of the most important fruits in the world due to its versatility [1–3]. It is also an easily cultivable fruit that can be stored and transported with ease. Since 2014, apple production in Morocco has grown by 6.5% in a single year, placing the country 18th among the major apple-producing nations in 2019, according to the Food and Agriculture Organization of the United Nations (FAO) [4]. In 2021, Africa’s total apple production reached 3.54 million tonnes, with Morocco contributing 889,736 tonnes [5]. South Africa remains the continent’s top producer, followed by Morocco, Egypt, and Algeria.

One of the most important insect pests of apple is Cydia pomonella, commonly known as the codling moth, which is a major threat to apple cultivation worldwide [6–8]. This insect possesses the ability to cause significant harm to an entire orchard in a matter of days. Its diet primarily consists of fruits, necessitating the implementation of targeted control measures [9]. Given the impact of international trade and tourism on its widespread dissemination, apple growers worldwide must address this issue [10]. The key concern in fruit production is to ensure the delivery of flawless, high-quality fruits without any signs of damage caused by this pest [11,12]. Although, this pest originally emerged in central Asia, it has since extended its presence to North America, Europe, South Africa, Northwest Africa, China, and Australia [13,14].

Beyond the specific challenges faced by apple growers, global agriculture is increasingly strained by population growth, which drives demand for agricultural products and leads to the expansion of cultivated areas [15,16]. Worldwide, agricultural production faces significant threats from biotic stresses, with recent estimates indicating that approximately 50% of agricultural losses are attributable to biotic factors, including pests and diseases, with insect pests alone accounting for 38% of total losses [17]. According to the FAO, up to 40% of crops are lost annually due to plant pests and diseases [18]. Timely and accurate pest control remains challenging, and over-reliance on agrochemicals can lead to environmental and health problems [19]. These losses cost the global economy over $220 billion annually, with invasive insects alone contributing at least $70 billion [20]. While pesticides are commonly used to protect crop productivity, their prolonged use poses serious environmental risks and health hazards, including cancer, respiratory diseases, and genetic disorders [21].

In Morocco and the rest of the countries, codling moth monitoring is typically conducted during periods of peak moth activity, which often coincide with the flowering stage of apple trees. This typically occurs in the spring and early summer, although the exact timing can vary depending on the region and the specific weather patterns [22]. Having accurate and timely data about the insects caught in traps can be useful for managing pest populations effectively. This information, such as the number and the types of insects, can provide insight into the size of the pest population and help farmers to identify the emergence period, determine the appropriate amount of pesticide to use, and assess the effectiveness of pest treatment. This data can be collected through regular monitoring of traps. It is an important aspect of integrated pest management.

Manual insect monitoring is a labor-intensive process that can be slow, tedious, and costly, particularly when the area is large. It also requires a skilled individual who can identify different insect species [23,24]. These factors make it attractive to automate the identification and counting process, as it helps save both time and resources. Additionally, traditional pest counting methods have a low temporal resolution, meaning getting feedback about pest populations takes a long time. This can be a problem when managing pests like the codling moth, as timely information is necessary to take immediate action. Automating the process could provide more timely and accurate data, improving the efficiency of pest management efforts [25,26].

Due to the aforementioned factors, both researchers and industry have shifted their focus to utilizing intelligent solutions, such as a mobile application that allows for identifying insects without needing an expert on-site to help users to identify the insects they have found [27]. In parallel, automated insect monitoring traps have been developed to improve the accuracy and efficiency of pest population monitoring in agriculture. These traps, essentially embedded systems consisting of hardware and software, use advanced sensor technology, microcontrollers, and telecommunication engineering to detect and record insects’ presence automatically. This allows for higher temporal resolution than manual counting and can be used to guide crop protection efforts. In recent years, embedded systems have become increasingly common in agriculture and are used with RGB cameras for remote surveillance [28–31]. These systems can link to wireless networks for orchard monitoring and offer both online and offline services to assist in decision-making for integrated pest management (IPM), which aims to keep pest populations under the economic injury threshold [32]. By continuously monitoring insect populations and coordinating interventions, such as insecticide spraying, farmers can save money and reduce the environmental impact of pest control efforts by applying insecticides only when necessary.

Beyond the hardware part, the automation of the insect counting process calls for the adoption of technologies like image processing, deep learning, and edge computing. Progress in machine vision and learning has enabled the use of deep object detection algorithms to effectively solve object detection challenges, including those in agriculture [33–35]. This is because insect counting can be considered a specific instance of object detection. Therefore, CNN-based object detectors can be effectively utilized. Many researchers are now studying image detection methods based on CNNs [36–40]. Within this field, the CNN-based YOLO (You Only Look Once) model stands out as especially suitable for applications that require both real-time response and high accuracy. One of YOLO’s key advantages is its ability to detect objects instantly, making it ideal for tasks demanding rapid processing. YOLO achieves reliable results by performing object detection and classification in a single pass, allowing for both speed and accuracy. This capability makes it particularly beneficial for agricultural applications like insect counting, where timely and precise detection is essential for effective pest management. Furthermore, YOLO’s design offers resilience to variations in object size, orientation, and partial occlusion, which adds to its adaptability in complex environments. Since its initial release, YOLO has evolved through twelve versions, each enhancing detection performance: YOLOv1 [41], YOLOv2 [42], YOLOv3 [43], YOLOv4 [44], YOLOv5 [45], YOLOv6 [46], YOLOv7 [47], YOLOv8 [48], YOLOv9 [49], YOLOv10 [50], YOLOv11 [51], and YOLOv12 [52].

To address the challenges of pest management in apple orchards, particularly against the codling moth (Cydia pomonella), this study aims to develop and validate an automated insect detection and monitoring system using deep learning and IoT technologies. Leveraging the YOLO model, renowned for its real-time detection capabilities, the proposed system enables timely and accurate monitoring of pest populations, supporting Integrated Pest Management (IPM) strategies. Through the integration of advanced image processing and edge computing, this solution not only enhances detection accuracy but also minimizes labor and environmental impact by ensuring insecticides are used only when necessary. This system represents a significant step forward in sustainable agricultural practices by optimizing pest control interventions, ultimately contributing to more efficient and eco-friendly apple production in Morocco and beyond.

The main contributions of this study are:

1. **Development of an Improved YOLOv10-m Model:** We present an enhanced version of the YOLOv10-m architecture, specifically optimized for accurate pest detection with reduced computational demands. This model is designed for efficient deployment on low-power devices such as the Raspberry Pi, thereby enabling real-time pest detection in resource-constrained agricultural environments while maintaining high detection accuracy and energy efficiency.
2. **Integration of IoT for Real-Time Monitoring:** The system incorporates IoT connectivity to enable real-time pest monitoring, automated data transmission, GPS-based trap localization, and threshold-triggered notifications. This integration provides actionable insights for farmers and supports more precise and timely interventions.
3. **Design of a Solar-Powered Smart Trap:** We propose and implement a solar-powered insect trap designed for remote apple orchards. The system is capable of autonomous, energy-efficient operation, with activity schedules aligned with codling moth behavioral patterns, ensuring optimal monitoring performance.
4. **Comprehensive YOLO Model Benchmarking:** A comparative evaluation of YOLO versions 5–12 was conducted under identical training and testing conditions. The improved YOLOv10-m model demonstrated superior detection accuracy, confidence stability, and computational efficiency.
5. **Promotion of Eco-Friendly IPM Practices:** By improving detection accuracy and enabling targeted pesticide application, the system supports Integrated Pest Management (IPM) strategies, reducing unnecessary chemical use and promoting sustainable, eco-friendly pest control practices.
6. **Identification of a Common Evaluation Flaw and Proposal of a Fair Comparison Method:** This study highlights a prevalent comparability error in prior research—comparing model results across studies without accounting for inconsistent training data or protocols. To address this issue, we recommend adopting a controlled evaluation methodology where all models are trained and tested under the same conditions, ensuring scientifically valid and reliable performance evaluation.

The remaining sections of the paper are organized as follows: Section 1 reviews the literature on traditional and automated insect trapping methods, focusing on challenges in tracking flying insects and the role of Integrated Pest Management (IPM) in sustainable agriculture. Section 2 outlines the materials and methods, detailing dataset preparation, object detection models, and the architecture of the proposed IoT-enabled pest monitoring system. Section 3 presents the experimental results, including evaluation metrics and real-time monitoring outcomes, demonstrating the system’s efficacy in pest detection. Finally, Section 4 summarizes the key findings and proposes future directions for advancing automated pest monitoring in agriculture.

## 2. Literature review

Effective monitoring of flying insect pests is crucial in agriculture due to their high mobility and potential for rapid spread across crop areas. The increased threat posed by these pests, especially for crops vulnerable to airborne invasions, has led to a range of strategies and devices designed to track and control insect populations in real time. This literature review examines the various trapping methods developed for field applications, highlighting both their advantages and limitations in the context of tracking mobile insect populations.

### 2.1. Traditional Trapping Methods

Traditional pest monitoring methods, including sticky traps and pheromone-based traps, remain widely used due to their low cost and ease of deployment. Sticky traps provide coarse estimates of insect population density by physically capturing insects on adhesive surfaces [53,54]. Pheromone traps, in contrast, are designed to attract specific insect species—most notably moths such as Cydia pomonella—through synthetic sex pheromones [55–57]. While these methods are effective for species-specific monitoring, they rely heavily on manual inspection, are labor-intensive, and do not support real-time decision-making. Moreover, they provide limited temporal resolution and are unsuitable for large-scale or continuous monitoring in commercial orchards.

### 2.2. Automated Trapping Systems

To overcome the limitations of manual monitoring, recent studies have explored automated trapping systems equipped with cameras and wireless communication modules, enabling continuous image acquisition and remote analysis of insect activity [58–60]. In these systems, images captured by smart traps are typically transmitted to centralized servers or cloud platforms, where they are analyzed either manually by entomologists or automatically using convolutional neural networks (CNNs) to detect and count insects such as moths, beetles, and fruit flies. Although CNN-based approaches significantly improve detection accuracy and automation, cloud-based processing introduces several practical limitations, including high bandwidth requirements, increased latency, dependence on stable network connectivity, and elevated energy consumption, which restrict their applicability in large-scale and remote agricultural environments.

From a sustainability and deployment perspective, continuous cloud-based image transmission and processing can be particularly inefficient for field applications. Consequently, recent research has shifted toward embedded and edge-based processing, where inference is performed locally on low-power devices integrated within the trap itself [61,62]. This edge AI paradigm reduces data transmission, lowers overall energy consumption, and improves system responsiveness, enabling near real-time pest monitoring. When combined with renewable energy sources such as solar power, embedded smart traps become more autonomous and environmentally sustainable. Therefore, the development of lightweight, efficient, and robust on-device insect detection models is essential for scalable and energy-efficient automated trapping systems in precision agriculture [63].

### 2.3. Acoustic and Echo-acoustic Traps

Incorporating acoustic technologies, such as echo-acoustic traps, has introduced alternative approaches for insect monitoring by exploiting species-specific wingbeat frequencies. These systems typically employ infrared light sources and Fresnel lenses to capture wingbeat signals, enabling species identification based on acoustic characteristics [64,65]. While echo-acoustic traps can distinguish certain insect species under controlled experimental conditions, their performance is often degraded in real-world agricultural environments due to background noise, overlapping wingbeat frequencies among species, and environmental variability. As a result, such systems are generally unsuitable for high-precision monitoring of economically important pests, including codling moths, in orchard settings.

### 2.4. Challenges in tracking flying insects

The high mobility of flying insects poses unique challenges in trapping and tracking efforts. Unlike ground-dwelling pests, flying insects can cover large distances rapidly, making it difficult to monitor their population dynamics and movement patterns accurately [28,66]. Environmental factors such as wind speed, temperature, and humidity further influence insect flight behavior, making real-time tracking essential for adaptive pest control.

### 2.5. Integrated Pest Management (IPM) and future directions

In the context of Integrated Pest Management (IPM), combining smart traps, remote sensing technologies, and real-time data analysis offers a promising approach to improving pest tracking capabilities. Integrating automated traps with decision-support systems enables farmers to receive timely alerts and actionable insights on pest activity, facilitating more precise control measures [1,67]. Research continues to focus on refining AI-driven systems for species identification, enhancing trap durability, and improving cost-effectiveness for wider adoption in various agricultural settings.

### 2.6. Recent advances in edge AI for pest detection

Recent developments in edge computing and lightweight deep learning models have demonstrated significant progress in achieving real-time pest detection on resource-constrained devices. A 2025 study introduced Tiny-LiteNet, a lightweight CNN optimized for Raspberry Pi 5 deployment, achieving 98.6% accuracy with only 1.2 MB model size and 80 ms inference time, enabling autonomous operation without cloud dependency [68]. Similarly, edge-based architectures combining 5G connectivity with IoT sensors have reduced latency by over 60% while improving data throughput by 40%, making them highly suitable for time-sensitive pest detection tasks [69]. Recent YOLO-based implementations have further advanced this field: an improved YOLOv10n model (YOLO-YSTs) achieved real-time pest detection on edge computing platforms for yellow sticky trap monitoring [61], while another YOLOv10-based system detected black aphids with 89.1% mAP50 accuracy and integrated mobile applications for farmer notifications [70]. These edge AI solutions address critical deployment constraints by performing local inference, reducing bandwidth requirements, and enabling autonomous operation in remote agricultural environments where stable internet connectivity is unavailable.

### 2.7. Proposed solar-powered edge AI Trap with IoT integration

To advance current pest management strategies, this research introduces a novel pheromone trap optimized for tracking codling moths in apple orchards, equipped with an improved YOLOv10-m model to achieve high-precision insect detection. Our enhanced YOLOv10-m architecture incorporates targeted modifications—including C3 blocks for comprehensive feature extraction, ConvTranspose2d layers for improved upsampling, and optimized CBS blocks replacing ScDown modules—that collectively enhance detection accuracy while maintaining computational efficiency suitable for edge deployment.

This upgraded system incorporates a Raspberry Pi 4B embedded platform that processes images locally on-site, eliminating cloud dependency and enabling autonomous operation in remote agricultural environments. The improved YOLOv10-m model achieves superior performance with 27.79B FLOPs and 19.52M parameters—representing a 13% reduction in computational operations compared to the standard YOLOv10-m—while maintaining high detection accuracy and confidence stability. The entire trap system, including the Raspberry Pi and camera module, is powered by a 12W solar panel paired with a 1820mAh rechargeable battery, ensuring energy-efficient continuous operation. Intelligent power management through PiJuice HAT schedules the system to activate twice daily—at 8:00 AM and 6:00 PM—coinciding with peak codling moth activity periods, thereby maximizing detection effectiveness while minimizing energy consumption for multi-day autonomous operation.

Once insects are detected and processed locally, both quantitative data (insect counts, GPS coordinates) and visual evidence (detection images) are transmitted in real time via cellular connectivity (SIM7000E module) to the Ubidots IoT platform, enabling comprehensive field monitoring without Wi-Fi infrastructure requirements. This platform provides farmers with timely and actionable insights through web and mobile dashboards, displaying pest population trends, trap geolocalization on interactive maps, and time-series analytics. When insect counts exceed predefined thresholds, automated alerts are immediately sent via email or SMS, prompting targeted intervention only when necessary. By correlating computational efficiency metrics (FLOPs, parameter counts) with practical deployment requirements (inference time, energy consumption), and by enabling precise, threshold-based pesticide application, this approach aligns with Integrated Pest Management (IPM) principles, promoting environmental sustainability through minimized chemical usage while maintaining effective pest control.

While similar initiatives have advanced intelligent trap development for insect detection and pest attack prediction, many lack essential features that limit their functionality (Ahmed et al. [71], Teixeira et al. [72], Hadi et al. [73], Mira et al. [74], Gao et al. [75], and Liu et al. [76]). For instance, some systems do not have internet connectivity, preventing remote data transmission and requiring farmers to be physically present in the field to retrieve information. Others rely solely on Wi-Fi networks, limiting their deployment in remote areas where such infrastructure is unavailable. Additionally, some systems lack geolocation capabilities, making it difficult to identify high-risk areas precisely and hampering timely intervention efforts. Many solutions also lack automated notification systems, requiring constant monitoring by the farmer. Our system effectively addresses these limitations by incorporating cellular-based internet connectivity, GPS mapping features, and threshold-based automated alerts, significantly enhancing the efficiency of pest monitoring and intervention. This comprehensive approach empowers farmers to protect their crops more effectively, reducing both labor and resource costs associated with traditional pest control methods.

This integrated system addresses critical gaps identified in current literature:

1. It eliminates cloud processing latency and bandwidth limitations through local edge inference;
2. It operates autonomously in remote locations via solar power and cellular connectivity, unlike Wi-Fi-dependent systems;
3. It provides comprehensive monitoring features including GPS localization, automated notifications, and visual verification, which many existing solutions lack;
4. It demonstrates the practical correlation between model optimization (reduced FLOPs/parameters) and field deployment viability (energy efficiency, real-time processing).

Collectively, these innovations not only enhance the accuracy and responsiveness of pest management but also establish a scalable, field-ready solution that supports sustainable agricultural practices through optimized resource use and reduced environmental impact.

## 3. Materials and methods

### 3.1. Dataset

To build and validate our insect pest detection system, we collected data from three primary sources. The first source consists of field and laboratory data obtained using pheromone traps deployed in apple orchards to monitor pest populations. This dataset was further enriched through collaboration with the team of Akroute et al. [77] at the National Institute of Agricultural Research in Morocco. Their study aimed to identify associations between apple cultivar characteristics and susceptibility to apple borer infestation, with the broader goal of supporting sustainable pest management strategies. As part of their methodology, the insect was reared under controlled laboratory conditions and monitored in the field using pheromone traps. The images they provided of the apple borer substantially contributed to the diversity of our training dataset.

The second primary source was the database utilized by Suto [78]. Additionally, we collected images from various online databases and search engines, including Bing, IPM Images, Google, Lepiforum, iStock, the EPPO Global Database, and Flickr.

Importantly, the dataset intentionally includes a combination of real-time trap images and habitus-style images, in which the insect is shown with its wings fully spread. The inclusion of habitus images was a deliberate design choice to improve the robustness of the detection model. In real trapping conditions, insects frequently become stuck to adhesive surfaces with their wings spread, folded, or partially distorted by glue. Such deformation alters the insect’s appearance compared to natural resting postures and can hinder reliable detection. Consequently, habitus images complement real trap-acquired images by providing clear morphological cues, thereby enhancing model generalization under realistic field conditions.

The resulting dataset is comprehensive, containing images of the target insect captured across a wide range of geographic regions where it is commonly found. This geographic and environmental diversity enhances the model’s ability to generalize and improves its robustness in real-world agricultural settings by simulating a variety of conditions.

**Fig 1:**
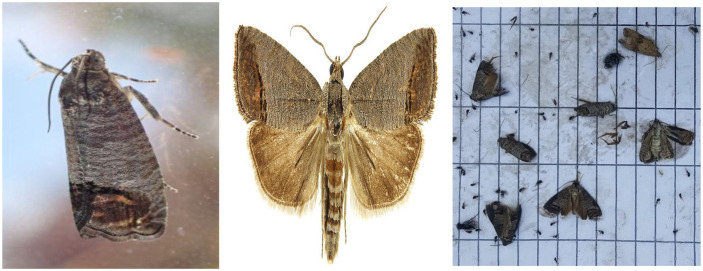
Sample images from the dataset for insect pest detection.

**Fig 2:**
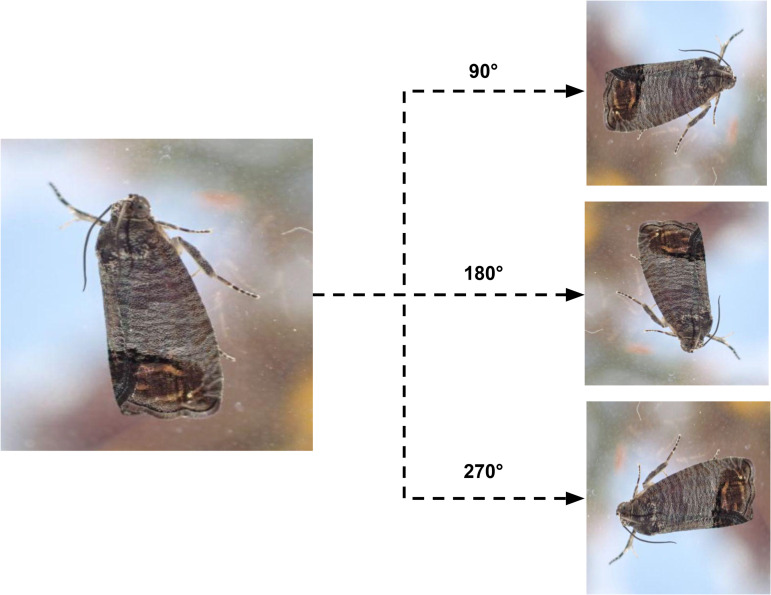
Augmented insect pest images using geometric transformations.

For consistency, all images were resized to a standardized 640x640 pixel dimension, and a few representative examples of these collected images are displayed in Fig 1. Since deep learning models typically benefit from larger datasets, we implemented data augmentation to increase the variety and number of samples. This technique reduces the risk of overfitting that smaller datasets might present. Specifically, geometric transformations—such as rotating each image at 90°, 180°, and 270°—were applied, resulting in three new images per original, increasing our dataset to a total of 1,011 images containing a total of 2,724 annotated insect instances, as illustrated in Fig 2.

**Fig 3:**
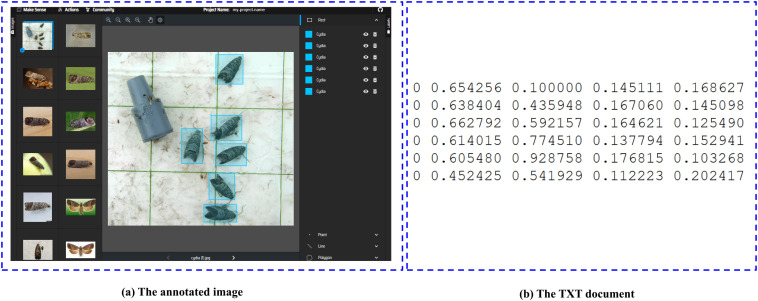
Image annotation process for training data using Makesense platform.

Image annotation is essential to preparing data for deep learning, as it involves identifying significant features and labeling them appropriately, which supports supervised learning. Annotated data allows the model to recognize and learn patterns in the imagery. In this study, we employed the MakeSense online platform [79] for annotation, streamlining the process and ensuring precise labeling of the dataset features. This process, depicted in Fig. 3, strengthened the dataset’s quality, providing a solid foundation for training the deep learning model and enhancing the system’s detection performance.

### 3.2. Object detection models

#### 3.2.1. YOLOv10 for object detection.

**Fig 4:**
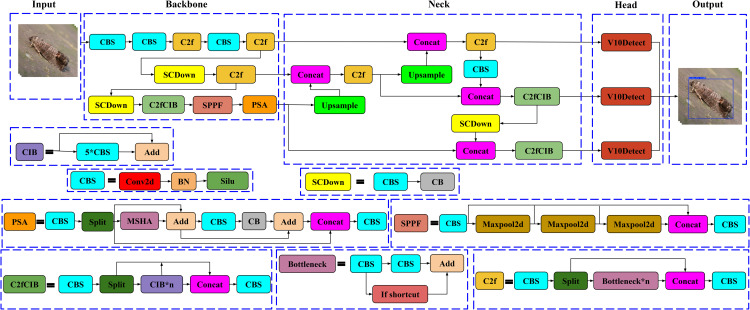
Overview of the YOLOv10 architecture, Highlighting Backbone, Neck, and Head components.

YOLOv10 builds upon YOLOv8’s architecture, with significant enhancements that improve detection accuracy and computational efficiency, especially for real-time applications. Key modifications in YOLOv10’s design include replacing and adding specific blocks to the Backbone and Neck, making it particularly adept at handling complex scenes with varied object sizes. An overview of the structure of the YOLOv10 model is shown in Fig 4.

- **Backbone**: The backbone of YOLOv10 is derived from YOLOv8 but includes several critical upgrades:
  - **ScDown (Scaled Convolutional Downsampling)** replaces the standard CBS blocks (Conv2D + Batch Normalization + SiLU) from YOLOv8, enabling effective downsampling with minimal information loss, thus improving efficiency and reducing computational load.
  - **C2fCIB (Cross Stage Partial Cross Iterative Block)** substitutes the C2f block from YOLOv8. This advanced block iteratively refines feature maps, allowing for detailed feature representation across stages and reducing redundancy, which enhances the detection of small and overlapping objects.
  - **PSA (Partial Self-Attention)** is added to the end of the backbone, enabling selective focus on critical spatial regions. This mechanism enhances feature refinement without significantly increasing computational cost, allowing YOLOv10 to maintain high accuracy even in complex environments.
- **Neck:** The neck in YOLOv10 is optimized for multi-scale feature fusion, incorporating additional components to improve small object detection:
  - **C2fCIB** is also included in the neck to further refine features, particularly for detecting small objects. By preserving fine-grained feature details, C2fCIB ensures that even small objects are accurately represented, facilitating improved detection performance.
  - **SCDown (Scaled Convolutional Downsampling)** is utilized in the neck as well, where it reduces the resolution of feature maps while retaining essential information. This setup supports faster processing and enhances the network’s ability to detect objects across varied scales.
- **Head**: In YOLOv10, the **V10Detection** module replaces the previous “Detection” head found in YOLOv8. This anchor-free detection approach simplifies object localization by focusing on center-based bounding box predictions, eliminating the need for predefined anchor boxes. V10Detection also employs a dual-label assignment strategy, combining one-to-one and one-to-many matching, which enhances detection precision and reduces the need for Non-Maximum Suppression (NMS) during inference, thus lowering latency.

With these enhancements, YOLOv10 achieves a balanced approach that ensures both high accuracy and speed, making it ideal for deployment on resource-constrained devices such as embedded systems and edge platforms. These upgrades enable YOLOv10 to effectively handle small objects, complex backgrounds, and varying object scales, making it particularly suitable for applications in fields like precision agriculture, surveillance, and autonomous navigation.

#### 3.2.2. Adding C3 and ConvTranspose2d to YOLOv10.

**Fig 5:**
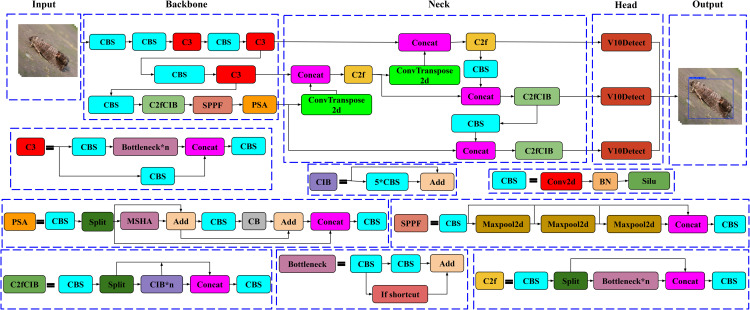
Overview of the improved YOLOv10 architecture with C3 and ConvTranspose2d enhancements.

In this enhanced YOLOv10 architecture, several targeted changes have been made to boost feature extraction and upsampling capabilities, essential for achieving high accuracy and confidence in detecting and identifying intricate objects, such as insects. Key modifications include the replacement of C2f with C3 in the backbone, ScDown with CBS, and the introduction of ConvTranspose2d in the neck. These changes improve the network’s ability to retain detailed spatial information and handle varied object scales effectively. The improved YOLOv10 model architecture, including modifications like C3, CBS, and ConvTranspose2d integration, is illustrated in Fig 5.

- **C3 Block in Backbone:** The C3 block has replaced the original C2f in the Backbone to enable more comprehensive feature extraction. Unlike C2f, the C3 block provides a deeper level of detail, essential for capturing subtle textures and patterns necessary for accurate object identification.
- **ConvTranspose2d in Neck:** To improve upsampling, ConvTranspose2d has replaced the standard Upsample layers in the neck. This learnable upsampling method allows for better reconstruction of spatial details in feature maps, enhancing localization accuracy, especially for small objects.
- **Replacing ScDown with CBS:** All instances of ScDown in the architecture have been replaced with CBS (Conv2d + Batch Normalization + SiLU). This substitution stabilizes the downsampling process while maintaining essential feature details, which supports accurate identification of small or complex objects by ensuring less information loss through the layers.
- **C2fCIB Block for Feature Refinement:** The C2fCIB block remains in use within both the Backbone and Neck sections due to its iterative refinement capabilities. It captures intricate structural details, especially valuable for objects with subtle, complex features, enhancing the model’s precision.

To evaluate the feature extraction capabilities of different blocks, three separate models were created: Model C2fCIB (*Input* ⇒ *C2fCIB* ⇒ *Conv* ⇒ *C2fCIB* ⇒ *Output*), Model C2f (*Input* ⇒ *C2f* ⇒ *Conv* ⇒ *C2f* ⇒ *Output*), and Model C3 (*Input* ⇒ *C3* ⇒ *Conv* ⇒ *C3* ⇒ *Output*). The images generated by each model highlight notable differences in their ability to capture detailed characteristics of the insect.

**Fig 6:**
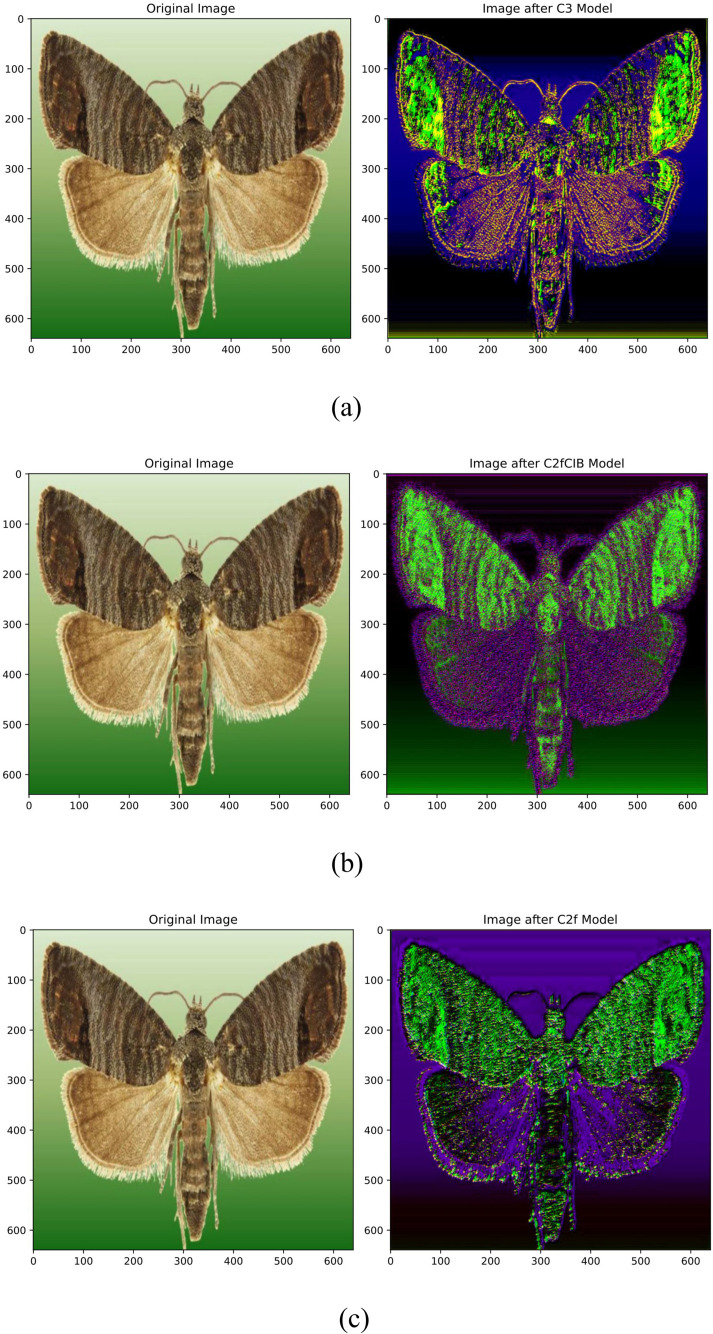
Comparison of feature extraction in different models: **(a)** C3 block, **(b)** C2fCIB block, and **(c)** C2f block.

- **C3 Block (**Fig 6a**):** The model using C3 blocks demonstrated superior detail extraction, capturing intricate textures and patterns, especially around the edges and finer structures of the insect. This level of detail makes C3 particularly effective for complex tasks that require high accuracy in recognizing subtle features.
- **C2fCIB Block (**Fig 6b**):** The model with C2fCIB blocks also captured detailed features well, though with a slightly different focus. C2fCIB is effective in iterative refinement, preserving intricate structures within the image. This block retains essential details, providing clear contrast between different textures, which aids in precise identification.
- **C2f Block (**Fig 6c**):** The model with C2f blocks displayed a lower level of detail compared to C3 and C2fCIB. While it successfully captures basic features, the fine textures and subtle patterns are less pronounced, making it less effective in applications requiring detailed differentiation of object features.

### 3.3. Architecture and Mechanism of the Proposed Insect Trap

**Fig 7:**
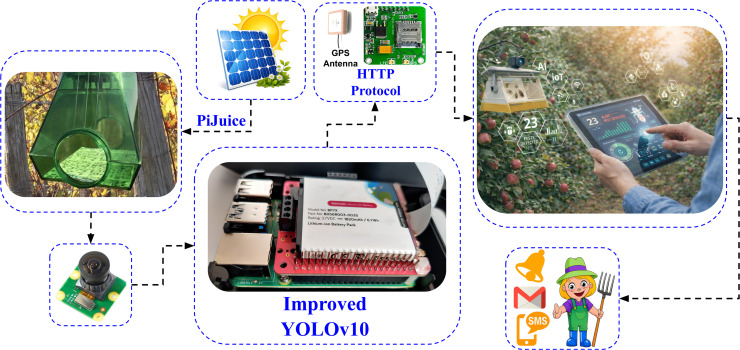
Functional overview of the IoT-based smart insect trap.

The architecture of the proposed insect trap combines pheromone-based attraction with advanced image processing and IoT integration, enabling real-time monitoring and data transmission of insect activity. The system, illustrated in the Fig 7, is designed to function autonomously in the field. It captures and processes insect images, then transmits relevant data to a central IoT platform for streamlined monitoring and management.

The key components of the system include:

- **Pheromone-Based Trap:** The trap uses pheromones to attract specific insect species, such as the codling moth. Pheromones ensure that only the target pests are drawn into the trap. This selective attraction minimizes the capture of non-target species, thereby enhancing the relevance of the data collected.
- **Camera Module:** Inside the trap, the camera module captures high-resolution images of the insects, providing clear input data for detection. With a resolution of 2592x1944 pixels and a 5-megapixel sensor, it ensures detailed imagery that enhances detection accuracy. Positioned at a fixed angle, the camera maintains consistent image quality, essential for reliable insect identification and counting.
- **Processing Unit with Improved YOLOv10 Integration:** The core of the processing system is a Raspberry Pi 4B (with 2GB RAM) configured to locally process images using an enhanced YOLOv10 model. This model is fine-tuned for precise insect detection, enabling accurate identification and counting of insects in real-time based on the captured images. By performing image processing locally, the Raspberry Pi sends both the insect count and the detection image to the IoT platform. This approach ensures that essential visual evidence is available along with quantitative data, providing more context for monitoring insect activity while managing bandwidth effectively.
- **Data Transmission via HTTP Protocol:** Processed data, including insect counts, detection images, and trap location coordinates, is sent to the IoT platform using HTTP via a SIM7000E module with cellular connectivity. This enables the trap to function without Wi-Fi, ideal for remote areas. By transmitting compact data packets, the system ensures efficient, real-time updates without network strain, providing timely information for monitoring.
- **IoT Platform and Farmer Notification:** The Ubidots IoT platform serves as the data visualization hub, where farmers and agricultural managers can access a dashboard for real-time pest monitoring. The platform stores historical data, enabling trend analysis and insights into insect behavior over time. When insect counts surpass a specified threshold, an alert is automatically sent to the farmer’s mobile device, prompting timely intervention. This threshold-based notification system supports Integrated Pest Management (IPM) by ensuring that pesticides are only applied when necessary, reducing environmental impact and operational costs.
- **Solar Power and Battery Backup:** A 12W (6V, 2A) solar panel powers the trap sustainably, paired with a 1820mAh rechargeable battery that ensures continuous operation during low sunlight. The PiJuice HAT optimizes power usage by scheduling the Raspberry Pi to activate twice daily—at 8 am after sunrise and at 6 pm, just before sunset—coinciding with the onset of codling moth activity. Codling moths typically begin flying and mating shortly before sunset and continue into the night under suitable environmental conditions, avoiding direct sunlight and high temperatures. Additionally, the activation schedule of the Raspberry Pi adjusts throughout the year to stay aligned with seasonal variations in codling moth activity. This setup maximizes energy efficiency, allowing the system to operate autonomously with minimal power consumption.

**Fig 8:**
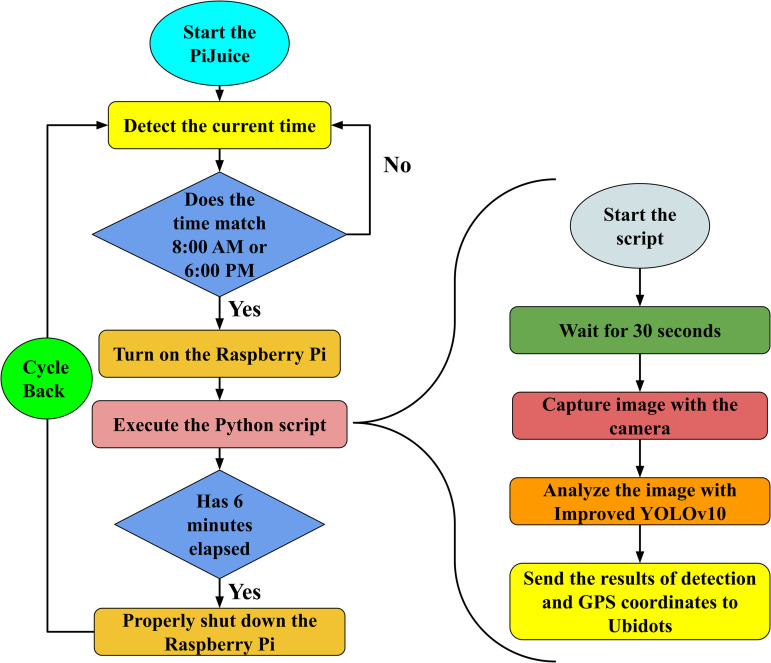
Workflow of the proposed insect trap system.

To illustrate the workflow of the system, Fig 8 shows the sequence of operations, from detecting the current time to capturing insect images and transmitting the results. The workflow begins with the PiJuice module, which starts and detects the current time. If the time matches the preconfigured intervals, specifically 8:00 AM or 6:00 PM, the Raspberry Pi is activated. Once activated, the Raspberry Pi executes a Python script that includes a brief delay to stabilize hardware components, such as the SIM7000E module and the camera module. Following this initialization, the system captures an image using the camera module and processes it using an improved YOLOv10 model for insect detection. The detection results, along with GPS coordinates, are then transmitted to the IoT platform via the SIM7000E module. After six minutes, the Raspberry Pi is powered off to conserve energy, and the system enters standby mode to await the next scheduled wake-up. Although the script itself typically takes less than three minutes to execute, the total duration is extended to six minutes to provide a buffer for unexpected delays or issues during execution.

In summary, the proposed insect trap leverages pheromone-based attraction, real-time image processing with improved YOLOv10, and IoT connectivity to deliver an autonomous, field-ready solution for pest monitoring. Solar power and intelligent scheduling enhance energy efficiency, while cellular data transmission enables remote deployment without Wi-Fi. By providing both quantitative insect counts and visual evidence, the system supports proactive pest management, aligning with Integrated Pest Management (IPM) principles to optimize pesticide use and reduce environmental impact.

## 4. Results

### 4.1. Experimental environment and evaluation metrics

In training the improved YOLOv10-m model (medium version, chosen for its balanced speed and accuracy) for insect pest detection, careful dataset preparation was essential. The labeling phase ensured precise annotations for both the training and validation datasets, specifically identifying insect pests relevant to our study, such as the codling moth. The model was trained on Google Colab, utilizing an NVIDIA Tesla T4 GPU with 16 GB of memory, supported by CUDA version 12.2 and driver version 535.104.05. All images were resized to a standard resolution of 640x640 pixels to enhance processing efficiency and maintain consistency. Class information, including names and counts, was organized in a configuration file (data.yaml), with the dataset divided into 85% for training and 15% for validation. The model was trained for 100 epochs with a batch size of 16, allowing it to learn detailed features specific to insect detection.

The performance of the improved YOLOv10-m was evaluated using key metrics essential for assessing its suitability for real-world pest monitoring applications:

- **Precision (P):** Measures the accuracy of positive detections, representing the proportion of correct insect identifications among all detected positives.
- **Recall (R):** Indicates the model’s ability to identify all relevant insect instances, measuring the rate of correct predictions for actual insect occurrences.
- **Floating Point Operations Per Second (FLOPs):** A measure of computational efficiency, indicating the number of operations required for the model to process a single image. This metric helps assess the model’s suitability for deployment in resource-constrained environments.
- **Mean Average Precision (mAP):** Calculated at an Intersection over Union (IoU) threshold of 0.5 (mAP50) and across a range from 0.5 to 0.95 (mAP50-95). These metrics provide insights into the model’s effectiveness at accurately localizing and identifying insect pests at different confidence levels.

The formulas for precision and recall are as follows:


$$\text{Precision}=\frac{\text{TP}}{\text{TP}+\text{FP}}$$ (1)

$$\text{Recall}=\frac{\text{TP}}{\text{TP}+\text{FN}}$$ (2)

Where:

- **True Positives (TP)**: Correctly identified detections of codling moths.
- **False Positives (FP)**: Instances mistakenly classified as codling moths.
- **False Negatives (FN)**: Codling moths that were missed by the model.

The mean Average Precision (mAP) is calculated using the following formulas:


$$AP=\int_{0}^{1}P(R)dR$$ (3)

$$mAP=\frac{\sum_{i=1}^{N}AP_{i}}{\;N}$$ (4)

Here, AP (Average Precision) is derived by integrating precision (P) over the range of recall (R). mAP (mean Average Precision) represents the average of AP values across all object classes, with N denoting the total number of object classes being evaluated. This metric provides a comprehensive assessment of the model’s accuracy and consistency at various IoU thresholds.

**Fig 9:**
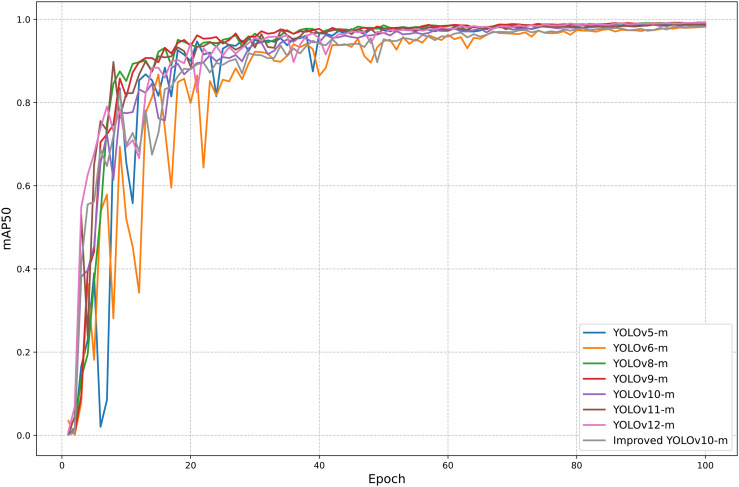
Workflow of the proposed insect trap system.

The training performance of the improved YOLOv10-m model, alongside seven other YOLO versions, is illustrated in Fig 9. The figure presents the mAP50 scores. Most models achieve high accuracy and converge rapidly, underscoring their learning efficiency. However, while these metrics provide valuable insights into training behavior, true model effectiveness must be validated on an independent test dataset. This evaluation step is crucial to ensure strong generalization and reliable performance in real-world codling moth detection within precision agriculture.

### 4.2. Experimental results

In this study, the improved YOLOv10-m model was evaluated against seven other YOLO versions (YOLOv5-m, YOLOv6-m, YOLOv8-m, YOLOv9-m, YOLOv10-m, YOLO-v11-m, and YOLOv12-m) to determine its effectiveness in detecting codling moths. Each model was tested on a consistent image containing codling moths, and detection results were assessed to compare accuracy, confidence stability, and average confidence levels.

**Fig 10:**
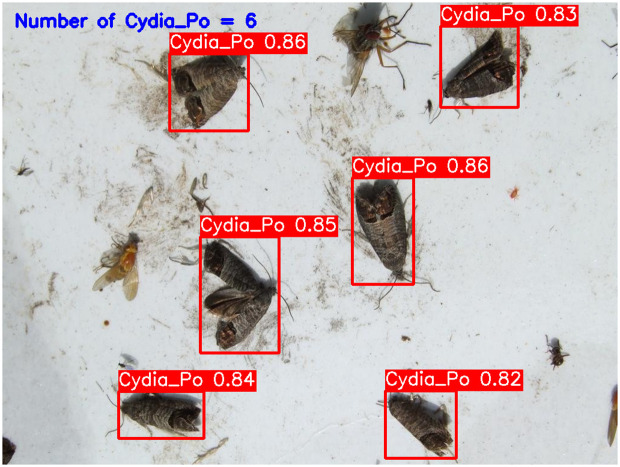
YOLOv5-m Results for Codling Moth Detection.

**Fig 11:**
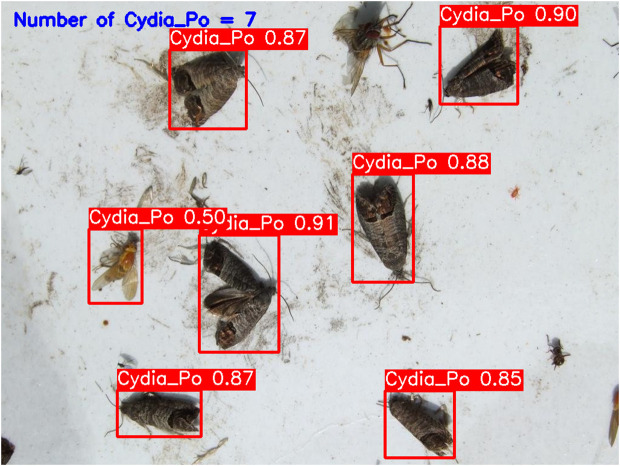
YOLOv6-m Results for Codling Moth Detection.

**Fig 12:**
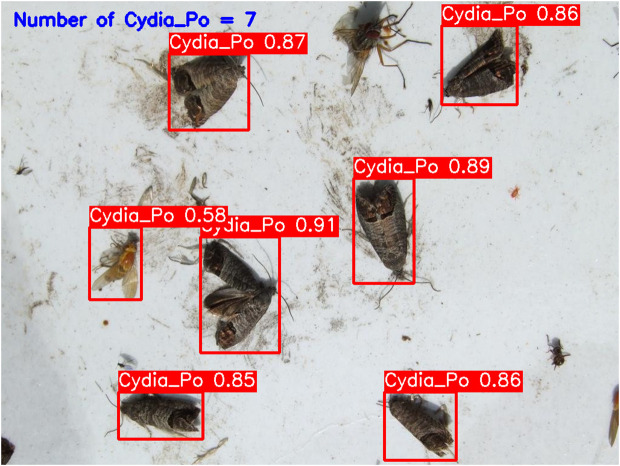
YOLOv8-m Results for Codling Moth Detection.

**Fig 13:**
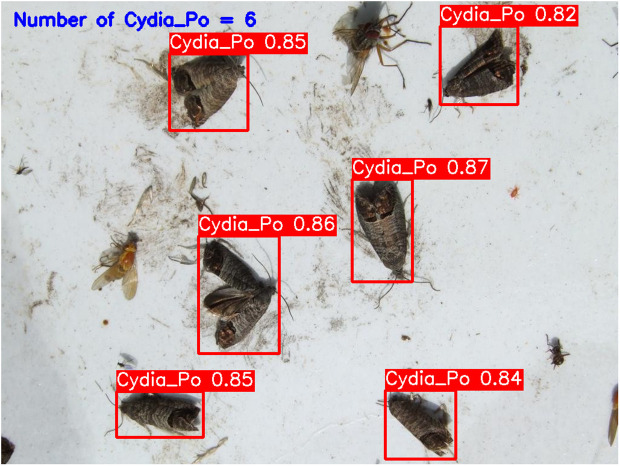
YOLOv9-m Results for Codling Moth Detection.

**Fig 14:**
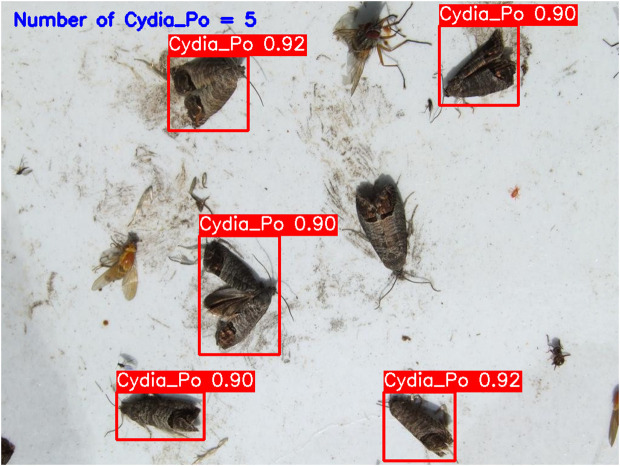
YOLOv10-m Results for Codling Moth Detection.

**Fig 15:**
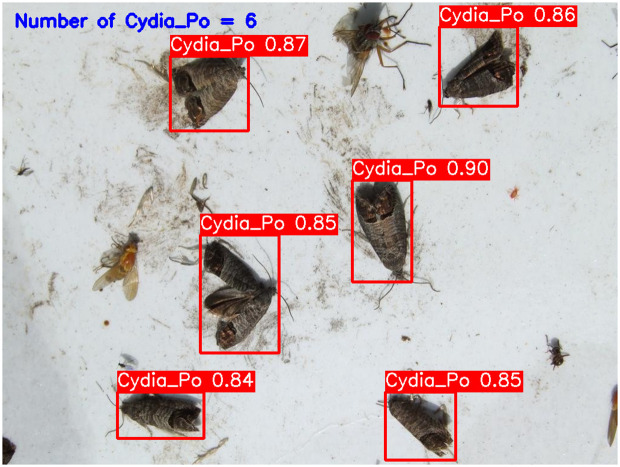
YOLOv11-m Results for Codling Moth Detection.

**Fig 16:**
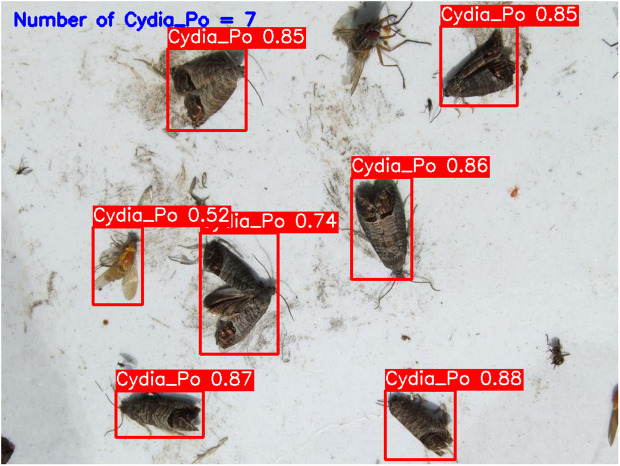
YOLOv12-m Results for Codling Moth Detection.

**Fig 17:**
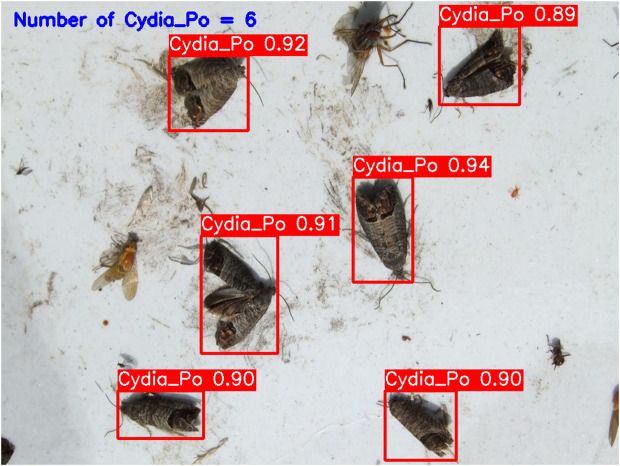
Improved YOLOv10-m results for Codling Moth detection.

- **YOLOv5-m (Fig 10):** This model accurately detected six codling moths with no false positives, achieving 100% precision and recall. Confidence scores ranged from 82% to 86%, with an average confidence level of 84%, indicating reliable detection performance.
- **YOLOv6-m (Fig 11):** YOLOv6-m identified seven objects, including one false positive, yielding an 86% precision and 100% recall. Confidence levels varied significantly, with scores as low as 50% (FP) and a high of 91%. The average confidence level was 81%, suggesting less stability in detection certainty.
- **YOLOv8-m (Fig 12):** Similar to YOLOv6-m, YOLOv8-m detected seven objects, including one false positive. The model had an average confidence level of 80%, with some detections as low as 58% (FP), indicating variability in detection confidence and stability.
- **YOLOv9-m (Fig 13):** This model accurately detected six codling moths with no false positives, achieving 100% precision and recall. Confidence scores ranged from 82% to 87%, with an average confidence level of 85%, showing stable and consistent detection performance.
- **YOLOv10-m (Fig 14):** YOLOv10-m detected five of the six target insects, achieving 100% precision but only 83% recall due to one missed insect. While confidence levels were consistently high, ranging between 90% and 92%, the missed detection suggests a trade-off in sensitivity for certain insect features. The model maintained an average confidence level of 91%, indicating overall reliability but with some limitations in detecting all instances of the codling moth.
- **YOLOv11-m (Fig 15):** YOLOv11-m achieved complete detection with six identified moths and no false positives, reaching 100% precision and recall. Confidence scores ranged from 84% to 90%, with an average confidence level of 87%, indicating consistent detection reliability.
- **YOLOv12-m (Fig 16):** YOLOv12-m detected seven objects, similar to YOLOv6-m and YOLOv8-m, including one false positive. This resulted in a precision of 86% and a perfect recall of 100%. Confidence scores ranged from 52% (false positive) to 88%, with an average confidence level of 79.57%. While the high recall indicates strong sensitivity, the low-confidence false positive highlights a slight inconsistency in detection reliability.
- **Improved YOLOv10-m (**Fig 17): The proposed improved YOLOv10-m model demonstrated the highest performance, detecting all six codling moths with no false positives, achieving both 100% precision and recall. It maintained a narrow confidence range from 89% to 94%, with an average confidence level of 92%, indicating exceptional consistency and reliability in detection.

The improved YOLOv10-m model demonstrated superior or comparable detection performance relative to other evaluated variants, achieving high detection accuracy, stable confidence scores, and elevated average confidence levels while maintaining reduced computational complexity (27.79 B FLOPs) compared with other versions, as summarized in Tables 1 and 2. These results highlight the model’s efficient parameter utilization and computational efficiency, making it particularly well suited for precise and consistent pest monitoring applications.

**Table 1:** Performance metrics of YOLO models for Codling Moth detection.

Model	Objects Detected	Precision (%)	Recall (%)	TP	FP	FN
YOLOv5-m	6	100	100	6	0	0
YOLOv6-m	7	86	100	6	1	0
YOLOv8-m	7	86	100	6	1	0
YOLOv9-m	6	100	100	6	0	0
YOLOv10-m	5	100	83	5	0	1
YOLOv11-m	6	100	100	6	0	0
YOLOv12-m	7	86	100	6	1	0
**Improved YOLOv10-m**	**6**	**100**	**100**	**6**	**0**	**0**

**Table 2:** Confidence and parameter metrics of YOLO models for Codling Moth detection.

Model	FLOPs (B)	Number of Parameters (M)	Average Confidence Level (%)	Min Confidence (%)	Max Confidence (%)
YOLOv5-m	32.08	25.06	84.33	82	86
YOLOv6-m	80.32	51.99	82.57	50 (FP)	91
YOLOv8-m	39.44	25.85	83.14	58 (FP)	91
YOLOv9-m	38.64	20.15	84.83	82	87
YOLOv10-m	31.91	16.4	90.8	90	92
YOLOv11-m	34.02	20.05	86.16	84	90
YOLOv12-m	35.35	20.13	79.57	52 (FP)	88
**Improved YOLOv10-m**	**27.79**	**19.52**	**91**	**89**	**94**

Furthermore, when deployed on a Raspberry Pi 4B edge computing platform, the proposed detection pipeline achieves an inference speed of approximately 0.83 frames per second (FPS), with an average power consumption of around 7 W during operation. This balance between detection reliability and resource efficiency underscores the model’s suitability for real-world agricultural deployments, where stable performance under constrained energy and hardware conditions is essential.

### 4.3. Controlled benchmarking strategy: Avoiding cross-study comparison bias

This study deliberately refrains from comparing the performance of the proposed model with results reported in other studies, as such cross-study comparisons can be scientifically unreliable. Our earlier work (Zarboubi et al. [80]) has emphasized the issue of comparison error, which arises when models are evaluated using results from unrelated studies without controlling for differences in datasets, training protocols, and experimental conditions. Notably, several prior works—such as Chen et al. [81], Liu et al. [82], Sun et al. [83], Suzauddola et al. [84], and Zhang et al. [85]—have fallen into this methodological trap by drawing comparative conclusions based on results obtained under different, and often incompatible, experimental settings. These uncontrolled discrepancies risk producing misleading interpretations, where apparent improvements are incorrectly attributed to architectural innovations rather than to evaluation inconsistencies.

To address this issue, this study compares the proposed model only with various YOLO versions by adopting a robust and controlled benchmarking strategy. All models were trained and evaluated under identical conditions using the same dataset and experimental setup. This approach ensures fair and valid comparisons, allowing for a more accurate and meaningful assessment of the actual improvements introduced by the proposed model.

An additional finding of this study further reinforces the central argument regarding the critical impact of dataset variability on model performance. When evaluating multiple YOLO versions under identical experimental conditions, YOLOv5 outperformed the more recent YOLOv12. This outcome confirms that model performance is not solely dictated by architectural advancements but is also heavily influenced by the nature and composition of the training data. For example, while YOLOv12 achieves superior results on the COCO dataset [86], this advantage did not carry over to the dataset used in this study. This clearly illustrates that altering the training data can lead to significant shifts in performance rankings among models. Therefore, these findings strongly validate the importance of methodological consistency—particularly the use of standardized datasets and evaluation protocols—to ensure fair, reliable, and scientifically sound model comparisons.

### 4.4. Outcomes of Real-Time pest monitoring and notification system

**Fig 18:**
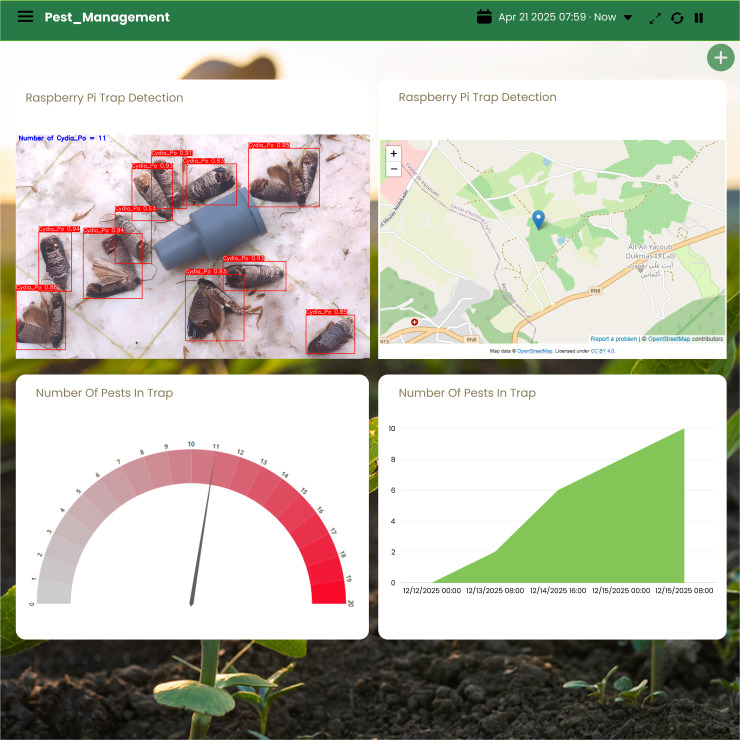
Illustrative example of a real-time pest monitoring dashboard accessed via a web interface. The figure was created by the authors for visualization purposes and is conceptually similar to IoT-based monitoring dashboards. It does not reproduce or adapt any proprietary interface elements and is provided for illustrative purposes only under the Creative Commons Attribution (CC BY 4.0) license. Base map and data from OpenStreetMap and OpenStreetMap foundation.

The integration of the improved YOLOv10-m model with an IoT platform has enabled efficient, real-time monitoring and management of codling moths (Cydia pomonella) in agricultural environments. As shown in Fig 18, the system captures and analyzes images from Raspberry Pi-based traps deployed in the field, detecting and labeling each insect with its class and confidence score. This automated visual identification allows farmers to verify pest counts at a glance and assess detection reliability. Alongside image analytics, the system displays the geolocation of each trap on an interactive map, enabling agricultural managers to identify infestation hotspots and respond with targeted intervention strategies.

**Fig 19:**
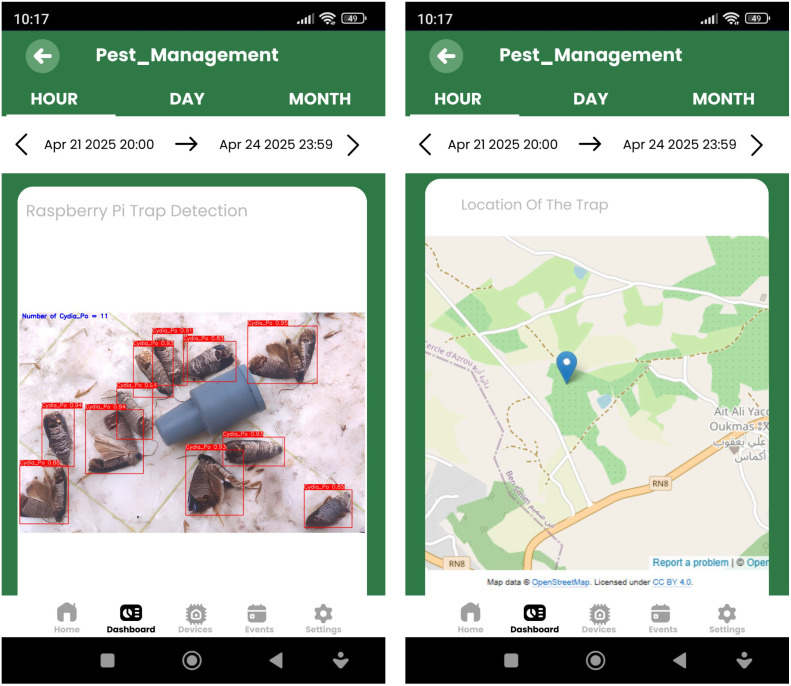
Illustrative example of a real-time pest monitoring dashboard accessed via a mobile application. This figure was created by the authors for visualization purposes and is conceptually similar to dashboards used in IoT-based monitoring platforms. It does not reproduce, adapt, or include any proprietary interface elements from third-party platforms. The figure is provided for illustrative purposes only and is published under the Creative Commons Attribution (CC BY 4.0) license. Base map and data from OpenStreetMap and OpenStreetMap foundation.

Quantitative tracking is further supported through dynamic data visualizations integrated into the IoT dashboard. A time-series chart records the evolution of pest populations over time, while a gauge meter provides an instantaneous view of the number of pests detected in the trap relative to a critical threshold. These tools offer actionable insights into pest population dynamics, helping farmers adapt control measures according to current trends. Moreover, the dashboard is accessible from both web and mobile devices, ensuring flexibility and ease of use. As illustrated in Fig 19, this cross-platform access allows users to monitor traps and make decisions remotely from anywhere and at any time.

**Fig 20:**
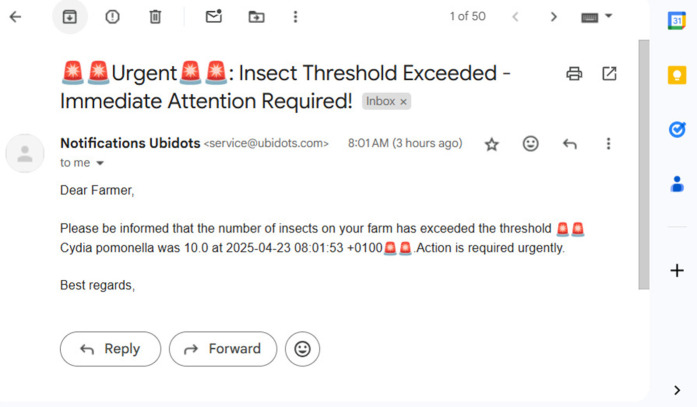
Email alert generated when pest count exceeds the configured threshold.

To complement real-time monitoring, the platform incorporates configurable event-based alerts. Users can set thresholds to automatically trigger notifications when pest populations exceed a predefined limit (e.g., 10 insects). When this condition is met, the system sends an urgent alert via email or SMS, as demonstrated in Fig 20. This automated warning system minimizes the need for manual surveillance and enables prompt action to prevent crop damage. For instance, on April 23, 2025, a detection of 10 codling moths triggered an immediate notification, emphasizing the platform’s effectiveness in supporting proactive and timely pest management.

The IoT platform’s features—including email alerts, image-based detections, trap location tracking, quantitative monitoring tools, a mobile app, and configurable event triggers—form a robust and user-friendly system for real-time insect monitoring and pest management. This integrated solution aligns with Integrated Pest Management (IPM) principles, supporting farmers in achieving efficient, autonomous pest control and promoting sustainable agricultural practices. Together, these advanced features enable farmers to respond promptly to pest threats, optimize pesticide use, and improve the long-term sustainability and profitability of their agricultural practices.

## 5. Conclusion and future work

This study presented a robust and energy-efficient pest monitoring system that integrates an improved YOLOv10-m model with IoT technology to address the challenges of real-time codling moth detection in apple orchards. The proposed solution achieved high detection accuracy, stable confidence scores, and low computational overhead, making it well-suited for deployment on low-power embedded platforms such as the Raspberry Pi. Leveraging real-time data transmission, automatic insect identification, and threshold-based alerting, the system enables timely and targeted interventions aligned with the principles of Integrated Pest Management (IPM).

By minimizing reliance on manual inspections and supporting precision pesticide application, the system promotes eco-friendly pest control practices that enhance both agricultural productivity and environmental sustainability. Its practical and field-deployable design—featuring solar-powered operation, cellular network connectivity, and mobile dashboard access—ensures adaptability in remote or infrastructure-limited agricultural settings.

**Fig 21:**
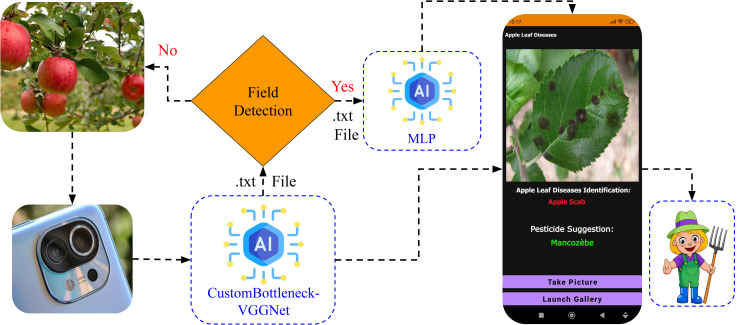
Proposed Pesticide Recommendation System for Apple Leaf Disease Management.

Future research directions will aim to further improve the system’s functionality, accessibility, and ecological impact through the following initiatives:

1. **Continuous data acquisition and incremental model learning:** The system will be extended to increase the number of seasonal training specimens through continuous collection of images captured by deployed traps throughout the year and their storage in *Firebase Cloud Storage*. This infrastructure will enable daily image acquisition across multiple seasons and deployment locations, facilitating the construction of a dynamically growing dataset that captures temporal, environmental, and regional variability. Such a framework will support periodic model retraining and incremental learning, thereby enhancing the system’s adaptability, robustness, and detection accuracy under evolving real-world field conditions.
2. **Development of a proprietary IoT platform and dashboard:** Efforts will be dedicated to designing and deploying an independent IoT-based monitoring platform. This will allow commercialization of the system at a competitive cost, ensuring accessibility to small and medium-scale farmers while eliminating reliance on external platforms.
3. **Design of a UV-light-based trap for greenhouse environments:** Unlike pheromone-based traps, UV-light solutions are more suitable for enclosed settings as they target pests already present inside. This will enable localized monitoring in greenhouses and support containment-oriented pest management.
4. **Integration of a Pesticide Recommendation System (PRS) for Leaf Disease Management:** The mobile application will be extended to include an intelligent decision-support module designed to monitor apple tree leaf health and recommend suitable pesticide treatments. This system will leverage a Multi-Layer Perceptron (MLP) model working in collaboration with our previously proposed CustomBottleneck-VGGNet model [80], which specializes in detecting various leaf diseases affecting apple trees. By analyzing daily disease detection results, the PRS will provide precise pesticide recommendations that minimize chemical usage and reduce environmental impact. This leaf disease detection system will function in parallel with the codling moth monitoring system, which determines the optimal timing for pest control interventions—together forming a unified and comprehensive smart farming solution for apple orchards (as shown in Fig 21).
